# A Codeveloped Web-Based Disability Disclosure Toolkit for Youth With Disabilities: Mixed Methods Pilot Evaluation

**DOI:** 10.2196/48609

**Published:** 2023-12-08

**Authors:** Sally Lindsay, Polina Kosareva, Nicole Thomson, Jennifer Stinson

**Affiliations:** 1 Bloorview Research Institute Holland Bloorview Kids Rehabilitation Hospital Toronto, ON Canada; 2 Department of Occupational Science and Occupational Therapy University of Toronto Toronto, ON Canada; 3 Institute of Health Policy, Management and Evaluation University of Toronto Toronto, ON Canada; 4 Lawrence Bloomberg Faculty of Nursing University of Toronto Toronto, ON Canada; 5 Hospital for Sick Children Toronto, ON Canada

**Keywords:** disability, disclosure, employment, vocational rehabilitation, youth and young adults, usability testing, qualitative, pilot study, co-design

## Abstract

**Background:**

Youth and young adults with disabilities experience many barriers in securing employment such as discrimination, inaccessible environments, and lack of support. Youth often need to decide whether and how they should disclose their need for accommodations to employers, which can help them to do their best at work. However, few evidence-based toolkits focusing on disability disclosure exist for youth with various types of disabilities. Supporting youth to develop self-advocacy skills is salient because they are an underrepresented and marginalized group in the labor market.

**Objective:**

The objective of this study was to conduct a pilot evaluation of a web-based toolkit to enhance disability disclosure for youth and young adults helping to advocate for their needs and request workplace accommodations.

**Methods:**

We conducted 2 in-person focus groups to codevelop a web-based disability disclosure toolkit, which was followed by a pilot evaluation with a pre-post survey. Primary outcomes focused on the relevance of the toolkit content, preliminary perceived impact on knowledge and confidence, and open-ended feedback on the usefulness of the toolkit. Secondary outcomes focused on effectiveness (ie, measures of self-determination).

**Results:**

A total of 14 youths with various types of disabilities took part in the study (aged 20-25 years; n=11, 78% female) including 3 who participated in the codevelopment focus group sessions and 11 youths who participated in the surveys. Our findings involved three main themes in the codevelopment sessions that included (1) disability disclosure and workplace accommodation experiences (ie, knowing when, whether, and how to disclose their disability and request workplace accommodations), (2) usefulness of the tool (ie, relatable content, format and design, and suggestions for further development), and (3) perceived impact of the toolkit (ie, navigating disclosure decisions and how to approach employers and develop other relevant employment skills). The survey findings showed that the majority of participants (10/11, 91%) reported that the toolkit increased or changed their knowledge or understanding of disability disclosure. Most participants (8/11, 73%) reported that the toolkit helped to increase their perceived confidence in their daily activities. The majority of participants (8/11, 73%) agreed or strongly agreed that the toolkit was easy to understand and comprehensive. Regarding the preliminary impact of the toolkit, participants did not demonstrate any significant improvements in self-determination (all *P*>.05).

**Conclusions:**

Our findings emphasize the importance of codeveloping a disability disclosure toolkit with youth to enhance its relevance for their needs. Our toolkit indicates preliminary potential as an educational resource for youth and young adults with disabilities as they search for and secure employment. Further research is needed to assess the impact of the tool with larger samples to understand the impact of workplace disability disclosure decisions for youth with disabilities.

## Introduction

People with disabilities, especially young adults, often experience lower rates of employment compared to people without disabilities [1]. The employment rate for Canadian youth with a moderate disability, aged 20-24 years, is 57% compared with 87% of youth without a disability [2]. Youth with disabilities are at risk of remaining unemployed after high school [3]. Many youth with disabilities often find it challenging to secure a job as they transition to adulthood [4-6]. The barriers that youth encounter in obtaining employment are often a result of societal and institutional barriers that they encounter such as stigma and discrimination, inaccessible workspaces, and lack of workplace accommodations [4,7,8]. Given that there are approximately 540,000 Canadians with a disability aged 15-24 years [9], it is imperative that they receive the necessary support and guidance to help enhance their employment success [10]. Focusing on youth and young adults with disabilities is salient because this developmental period is an optimal time to enhance positive behaviors while also fostering their work-based identities [5,10-12].

Supporting youth with disabilities regarding whether, when, and how to disclose their condition (ie, diagnosis, characteristics, or workplace needs) to an employer is important because it is necessary in order to receive workplace accommodations to help youth to perform their best [10,13-16]. Although workplace accommodations can help foster well-being and participation in the workforce, many people with disabilities are hesitant to disclose their condition for fear of stigma, discrimination, or job loss [4,6,17,18]. Indeed, research shows that only a small proportion of employees with disabilities disclose their needs to employers [19,20]. Not disclosing a disability can be somewhat worrying because working without needed accommodations can affect health, work productivity, and quality of life [21-23]. Nondisclosure is especially concerning among youth and young adults with disabilities because they are likely to lack work experience and more often work in precarious jobs and may be concerned about their future employment [3]. Additionally, youth with disabilities often have unique developmental needs, and workplace accommodation policies that are implemented for adults may be inappropriate for youth [10,19,24]. Thus, youth could benefit from additional support such as whether, when, and how they should consider disclosing their condition to their employer or potential employer [10]. Although many disability disclosure tools and interventions exist, they are targeted for adults [25-27], with a lack of attention to the needs of youth and young adults with disabilities as they enter their first employment experiences. Having a purposively built toolkit that is codeveloped with youth with disabilities may help to address these issues. The aim of our web-based toolkit was to enhance the skills of youth and young adults with disabilities and support them to advocate for their needs while also considering the benefits and potential disadvantages of disclosing their disability and requesting workplace accommodations [10]. Having disability disclosure supports could enhance opportunities to make the most appropriate decision. Disability disclosure outcomes could have a profound impact on self-confidence, career outcomes, and the development of relevant work-related skills [28]. Addressing such issues is critical because research shows that securing employment at an early age is a predictor of future successful employment in adulthood [29].

## Methods

### Objective

The objective of this study was to describe the codevelopment process of the toolkit and conduct a pilot evaluation of a web-based toolkit to help youth and young adults with disabilities to decide how and when to disclose their disability and ask for workplace accommodations.

### Ethical Considerations

This study received institutional research ethics board approval (#0162). All participants provided written consent (or e-consent via REDCap [Research Electronic Data Capture; Vanderbilt University] during the pandemic) prior to taking part in the study [30]. Participants who took part in the codevelopment sessions received a CAD $20 (US $14.47) gift card, and those who completed the surveys received a CAD $40 (US $29.14) gift card as a token of appreciation for their time, as recommended by our research ethics board. We anonymized and deidentified the data before analysis to protect the privacy and confidentiality of the participants.

### Design and Procedure

We conducted 2 in-person codevelopment focus group discussions to codevelop the web-based disability disclosure toolkit. After the toolkit was complete, a survey was conducted to assess the relevance of the toolkit content, preliminary perceived impact on knowledge and confidence, and overall feedback on the usefulness of the tool.

### Codevelopment Focus Group

Two codevelopment focus group discussions (with 3 youths with disabilities) lasting 2.5 hours each were conducted to explore participants’ experiences and perspectives of workplace disability disclosure in building the toolkit. This design is consistent with the development of toolkits and simulations [10,31]. These codevelopment sessions were facilitated by researchers who are certified in SIM-One simulations. The first session focused on building the simulation scenario content involving workplace disclosure decisions based on youth’s actual experiences, which was facilitated by a simulation educator. The second session (described in further detail below) involved piloting the disability disclosure toolkit content with 3 youths with disabilities who were asked for their feedback on the simulation content, its relevance, and any recommendations they had for further development [10].

### Development of the Web-Based Disability Disclosure Toolkit

Toolkits are a useful way to organize several knowledge translation strategies while educating and facilitating behavior change and outcomes [32,33]. Research shows that combining web-based resources with interactive knowledge translation strategies can increase the likelihood of successful outcomes in evidence-based practice, knowledge, skills, and behavior [34,35].

Our web-based toolkit [10], targeted for youth and young adults with a variety of disabilities who were interested and capable of paid employment, involved interactive and immersive learning tools including a PDF, a 5-minute animated video on workplace rights, a 5-minute articulate storyline based on the lived experience of disability disclosure decisions, and two 5-minute simulation videos showcasing scenarios of how youth could disclose their disability to an employer.

The toolkit was codeveloped with youth with disabilities, a knowledge-user advisory group, and evidence-informed content that was developed by members of our team [10] (also refer to Multimedia Appendix 1 for example content). The whole toolkit took the youth approximately 1-2 hours to review and was designed to be flexible in its use either reviewing it alone or in a group-based activity.

### Interactive PDF

The web-based PDF tool involved the following topics: what is disability disclosure and why it is important; things to consider before disclosing; how, when, and whether to ask for workplace accommodations; learning to self-advocate; knowing your workplace rights; and words of advice section (including youth and employer case studies) [10]. The tool was codeveloped with youth with disabilities, who helped to write some sections and also offered advice for the layout and graphic design. Youth also reviewed an earlier draft of our tool for usefulness and comprehensiveness, and their suggestions were incorporated into the final version. After our content was reviewed and approved by our team, it was assessed by our hospital’s health literacy committee [10].

### Articulate Storyline

A youth with a disability, who was a paid member of our team, scripted a storyline based on their own lived experience of looking for a job and returning to work after experiencing an acquired brain injury [10]. After they drafted their script, they shared it with the research team for feedback, which they incorporated into the final version along with input on the graphic design. They used Articulate 360 (Articulate Global Inc), a multimedia platform and e-learning authoring tool, which has a branching feature allowing participants to follow different routes depending on the choices they want to make, in addition to appropriate feedback.

### Animated PowToon Workplace Rights Video

Our previous research and needs assessments indicated that youth with disabilities needed more information about workplace rights [10]. Therefore, a youth with a disability, who was a paid member of our team, developed an animated video (using PowToon software [PowToon Ltd], a multimedia platform and e-learning authoring tool) that described workplace rights for young workers in a youth-friendly manner [10]. Next, they developed a script that was audio recorded along with the PowToon video software and relevant graphics.

### Disability Disclosure Simulation Videos

The toolkit involved 2 simulation videos that were represented by professional actors (ie, standardized patients). Simulations involve portraying real-life environments that are based on social situations based on real-world issues. They are often used within health care for skill development and learning to help enhance training and patient outcomes [36,37]. The development of the scripts for these simulations was informed by our previous research and needs assessments and codeveloped by 3 youths with disabilities. These developments were based on their actual lived experiences, which helped to enhance the relevance and authenticity [10]. The first simulation showed how an employer hiring a youth with a disability addressed disability-related assumptions and biases. This simulation involved a youth with a physical disability who was at the first job interview. The youth emailed the hiring manager in advance of the interview to ask if the building was accessible. The manager had limited experience in working with people who have a disability. The simulation illustrates how the employer and youth reacted to the situation [10]. The second simulation video was codeveloped with youth with disabilities based on their lived experiences. It illustrated how an employer, who recently hired a youth with a disability, addressed disability assumptions and biases. In particular, the simulation involved a youth with a nonvisible disability who was recently hired and completed the training and wanted to meet with the manager, who had limited experience working with someone who has a disability, to request a workplace accommodation. The simulation highlighted the reactions of the manager, showing both a less-than-ideal response followed by a more positive interaction [10].

### Survey Pilot Evaluation

We conducted a pre-post survey to assess the relevance and initial feedback of our web-based toolkit with the intention to examine the methods and procedures to test at a larger scale [38]. After consenting to participate, youth were asked to review the web-based toolkit (approximately 1 hour) remotely, on their own, via a password-protected portal. After viewing the toolkit, participants completed a web-based survey that a researcher sent via a REDCap link [30]. Study data were collected and managed using REDCap, a secure web-based software platform designed to support data capture for research studies hosted at our institution [30].

### Sample and Recruitment

For the codevelopment focus groups, data were collected in July and August 2019. For the pilot evaluation, data were collected from May 2021 to December 2022 and led by the first author with support from a research assistant. It is important to note that the data collection period for the pilot evaluation was interrupted by restrictions related to the COVID-19 pandemic. Using a purposive sampling strategy, aiming to recruit a variety of youth, participants were recruited through advertisements at a pediatric rehabilitation hospital and relevant community-based disability organizations and relevant social media platforms. We screened participants for inclusion with the following eligibility criteria: youth aged 15-29 years (based on the United Nations definition of youth) [39], able to read or write in English, have a disability (defined as impairments in body function or structure, activity limitations, and participation restrictions) [40], currently employed, enrolled in training, or seeking employment.

For our codevelopment sessions, we aimed to recruit 4-6 participants, which aligns with the recommendations for the optimal size for building toolkits and simulations [41,42]. For the pilot evaluation survey, we aimed to recruit 10-20 participants, which is considered appropriate for a pilot study to assess the usability of a web-based tool [43]. Some studies indicate that a sample size of less than 30 could be beneficial for exploring outcomes and initial impact and feasibility of web-based tools [15,44,45].

### Outcome Measures

Our primary outcome measures to assess the toolkit included the relevance of the content and perceived impact on knowledge and confidence related to disability disclosure [10]. Questions were adapted from the Community Impacts of Research-Oriented Partnerships (subscales on personal knowledge and development) and focused on whether participants increased or changed their personal understanding about the topic and whether they changed their beliefs and understandings with respect to an approach or group of people (ie, led to a new way of thinking or new perspective and altered ideas about how to approach an issue) [46]. These questions were measured on a 7-point scale (ie, not at all, a very small extent, to a small extent, to a moderate extent, to a fairly great extent, to a great extent, and to a very great extent) [46].

In regard to the relevance of the toolkit content, we drew on an adapted question from the toolkit evaluation questionnaire by Malik et al [47] that examined the comprehensiveness (ie, the extent to which the toolkit showed aspects of disability disclosure related to each specific tool in our toolkit) and relevance (ie, the extent to which toolkit was easy to understand for each of the components of the toolkit) of the information in the toolkit. This was measured on a 5-point scale (ie, strongly disagree, disagree, neither, agree, and strongly agree) [47].

In addition, we assessed the satisfaction of the toolkit by drawing on a survey from Kirkpatrick and Kirkpatrick [48]. The question asked is “How likely you change your behavior as a result of the toolkit?” which was measured on a 3-point scale (ie, somewhat agree, moderately agree, and agree a lot). We also asked “After reviewing this toolkit, will you disclose your disability to an employer?” and “Will you ask for workplace accommodations?” Responses included yes, no, or already doing this.

Other measures to assess the preliminary impact of the toolkit included Arc’s self-determination scale, a self-report measure assessing self-determination for adolescents with disabilities (ie, autonomy, self-realization, and psychological empowerment subscales) [49]. This measure has been used for youth with disabilities and has good test-retest reliability, internal consistency, and construct-related and criterion validity [49].

Other secondary measures included demographic measures such as age, gender, type of disability, assistive devices, education level, and type of work. We also had open-ended questions that asked youth about their thoughts on the toolkit, any suggestions they had, what parts they found most and least useful, and why. Secondary measures related to the qualitative data consisted of the focus group codevelopment sessions (eg, what youth liked most and least about the toolkit and any suggestions for further development).

### Data Analysis

Data were exported from REDCap [30] into SPSS (version 25; IBM Corp) for analysis. Descriptive statistics were used to describe sample characteristics and frequencies for categorical variables. We conducted 2-tailed *t* tests to assess the toolkit effects (comparing preintervention survey primary outcomes, time 1) and posttest data (immediately postintervention, time 2). A separate analysis was run for each outcome.

The analysis of the qualitative data from the focus group codevelopment sessions and the open-ended survey questions involved a directed content analysis approach [50]. Using an open-coding approach, 2 researchers independently read all the responses of the open-ended questions and developed preliminary codes while noting key patterns and trends [51]. Then, we met to compare and contrast the codes along with team discussions that helped to resolve any discrepancies in the organization of the codes and the development of the themes, which were revised in discussion with the research team. The first author, experienced in qualitative research, applied the final coding scheme to all of the qualitative data. Next, relevant quotes that represented each theme and subtheme were extracted. We used several strategies to help improve the rigor and trustworthiness of the findings including descriptive participant accounts and peer debriefing discussions among the research team who have expertise in occupational rehabilitation, youth with disabilities, and employment [52]. Additionally, notes were kept on analytical decisions made throughout the process. Finally, the team reflected on our own potential biases and how this may have impacted the development of the themes [52].

## Results

### Participant Characteristics

A total of 14 youths participated in this pilot study. Overall, 3 youths, aged 20-24 years with disabilities (2 with cerebral palsy and 1 with acquired brain injury) participated in the codevelopment focus groups. In total, 11 (9 women and 2 men) participants completed both pre-post surveys, ranging in age from 20 to 25 years (Table 1). Overall, 5 youths had cerebral palsy, 1 had spina bifida, 1 had autism, and 4 had other various types of disabilities (ie, mental health conditions, chronic pain, juvenile arthritis, and attention-deficit/hyperactivity disorder). In regard to the highest level of education, 1 youth was in high school, 2 had a high school diploma, 2 had a high school diploma, 6 had a college or other nonuniversity certificate, 2 had a bachelor’s degree, and 1 had a doctorate degree. Four youths used an assistive device (ie, wheelchair or walker). In regard to employment status, 5 worked part-time, 2 worked in a co-op or paid intern position, and 4 were currently seeking employment.

**Table 1:** Overview of participants in pilot evaluation of youth with disabilities (N=14).

Participant ID		Disability type	Sex	Assistive devices	Highest level of education	Employed
**Focus group participants**						
	1	Cerebral palsy	Male	Yes (wheelchair)	College	Part-time
	2	Acquired brain injury	Female	No	Bachelor’s degree	Full time
	3	Cerebral palsy	Female	Yes (wheelchair)	Master’s degree	Full time
**Survey participants**						
	4	Cerebral palsy	Female	No	College certificate	Part-time
	5	Mental disability	Female	No	Bachelor’s degree	Looking for work
	6	Spina bifida	Female	Yes (walker)	University certificate	Part-time
	7	Physical disability	Female	No	Doctorate degree	Co-op or internship
	8	Cerebral palsy	Male	Yes (ankle foot orthosis and wheelchair)	College diploma	Looking for work
	9	Juvenile rheumatoid arthritis	Female	No	High school diploma	Co-op or internship
	10	Cerebral palsy	Male	Yes (walker and stander)	High school diploma	Looking for work
	11	Cerebral palsy	Female	Yes (wheelchair)	College diploma	Part-time
	12	Cerebral palsy	Female	No	College diploma	Part-time
	13	Autism	Female	No	College	Looking for work
	14	Attention-deficit/hyperactivity disorder and learning disability	Female	No	Bachelor’s degree	Employed part-time

### Qualitative Findings

#### Overview

We developed three themes to describe the codevelopment sessions and pilot-testing that included (1) disability disclosure and workplace accommodation experiences, (2) usefulness of the tool (ie, relatable content, format and design, and suggestions for further development), and (3) perceived impact of the toolkit (see Multimedia Appendix 2 for an overview of themes and exemplar quotes).

#### Disability Disclosure and Workplace Accommodation Experiences

Participants shared their job interview and disclosure experiences to inform the content of the interactive parts of the toolkit. In the codevelopment focus group sessions of the toolkit, youth described the importance of knowing whether, when, and how to disclose their disability and request workplace accommodations. They explained that there are often many factors to consider such as whether the disability is visible or less visible to an employer. Youth with physical disabilities reported they often felt they had to address it upfront when meeting their employer for the first time, whereas youth with less visible disability could decide whether or not they wanted to disclose. The youth also considered the most optimal timing for requesting accommodations (if at all) including at the application stage, during the interview, or after they are hired. For example, a youth explained,

As someone with a very visible disability, I obviously don’t really have a choice about disclosure in a lot of situations. I’m disclosing pretty much the second I walk in the door.

Participant 1, male youth with cerebral palsy

Youth also contemplated how they would ask for accommodations. A youth described, “how do you present the accommodations that you need?; because there is kind of a subtle art to it” [participant 3, female youth with cerebral palsy]. For the youth with physical disabilities, they agreed that it was important to address any potential concerns that an employer had.

#### Usefulness of the Tool

Participants described their experience with using the toolkit, particularly the relatable content, the format and design, and suggestions for further development.

#### Relatable Content

Most youth expressed how relatable the toolkit content was and especially the case study examples, self-advocacy, words of advice sections, and simulation videos illustrating how to disclose to an employer and request accommodations. For example, a youth shared, “I liked the examples of youth stories. It’s great to see peer examples and stories that can be used in one’s own experience” [participant 4, female youth with cerebral palsy]. Youth appreciated learning how to plan what to say to their employer or potential employer.

#### Format and Design

Most of the participants reported enjoying the format and design of the toolkit and particularly how it was presented in a youth-friendly and appealing way. For example, a youth shared, “I feel like all the information was so important” [participant 6, female with spina bifida]. Some participants also described how they liked the interactive components and especially the videos and articulate storyline. A youth shared, “The interactive nature of toolkit is well done” [Participant 7, male youth with cerebral palsy].

#### Suggestions for Further Development

Some participants expressed that no changes were needed to the toolkit, while some others offered suggestions for further development of the toolkit that included having more employer-simulated conversations with sample responses. One youth described they would have liked more examples from youth sharing their employment experiences from a wider variety of employment situations. Some youth had specific comments related to the layout and design of the toolkit. For instance, 1 youth told us, “Have a section where you highlight the best takeaways and most important notes” [participant 8, male youth with cerebral palsy].

#### Perceived Impact of the Toolkit

Most participants explained how the toolkit would be helpful for them to navigate disclosure decisions and how to approach their employer to request workplace accommodations and develop other relevant employment skills. Youth appreciated how useful the toolkit content was for building their confidence in learning how to request workplace accommodations and how to have such conversations with their employer. A youth explained:

It helped me understand what skills I need to work on when preparing for job interviews, disability disclosure and accommodation request conversations. For example, it highlighted that I need to make my responses more clear and concise.

Participant 1, male youth with cerebral palsy

Another youth similarly shared, “It is useful to know what kind of accommodations are out there I could potentially ask for” [participant 9, female youth with juvenile arthritis].

### Quantitative Findings

Table 2 highlights the initial perceived impact of the toolkit. The majority of participants (10/11, 91%) reported that the toolkit increased or changed personal knowledge or understanding of the topic of disability disclosure (ie, n=2, 18% to a small extent; n=4, 36% to a moderate extent; n=2, 18% to a fairly great extent; and n=2, 18% to a great extent; Table 2).

**Table 2:** Preliminary perceived impact of the youth’s toolkit (n=11)^a^.

Preliminary perceived impact			Values, n (%)
**Increased or changed personal knowledge or understanding of the topic of disability disclosure**			
	Not at all	1 (9)	
	To a very small extent	0 (0)	
	To a small extent	2 (18)	
	To a moderate extent	4 (36)	
	To a fairly great extent	2 (18)	
	To a great extent	2 (18)	
	To a very great extent	0 (0)	
**Changed belief and understanding regarding intervention or approach or topic or a group of people**			
	Not at all	2 (18)	
	To a very small extent	0 (0)	
	To a small extent	2 (18)	
	To a moderate extent	1 (9)	
	To a fairly great extent	2 (18)	
	To a great extent	2 (18)	
	To a very great extent	1 (9)	
	Do not know	1 (9)	
**Perceived increased confidence in your professional or daily practice or activities**			
	Not at all	2 (18)	
	To a very small extent	0 (0)	
	To a small extent	1 (9)	
	To a moderate extent	1 (9)	
	To a fairly great extent	1 (9)	
	To a great extent	4 (36)	
	To a very great extent	1 (9)	
	Do not know	1 (9)	

^a^Note that numbers are rounded as per journal style.

The majority of participants (8/11, 73%) reported they had a changed belief and understanding regarding the intervention or topic (ie, n=2, 18% to a small extent; n=1, 9% to a moderate extent; n=2, 18% to a fairly great extent; n=2, 18% to a great extent; and n=1, 9% to a very great extent). Most participants (8/11, 73%) reported that the toolkit helped to increase their perceived confidence in their daily activities (ie, n=1, 9% to a small extent; n=1, 9% to a moderate extent; n=1, 9% to a fairly great extent; n=4, 36% to a great extent; and n=1, 9% to a very great extent).

Table 3 shows that the majority of participants (8/11, 73%) agreed or strongly agreed that the toolkit was easy to understand and comprehensive. For the part on how and when to ask for accommodations, 6 (54%) agreed and 5 (46%) strongly agreed. For the PowToon workplace rights video, 5 (45%) agreed and 4 (36%) strongly agreed it was easy to understand. Similarly, for the words of advice section, 4 (36%) agreed and 5 (54%) strongly agreed. For the articulate storyline section, 3 (27%) agreed and 7 (63%) strongly agreed. For the simulations, 4 (36%) agreed for simulation 1, 5 (45%) agreed for simulation 2, and 6 (54%) strongly agreed for both simulation 1 and 2. Finally, for the appendices and resources section, 2 (18%) participants agreed and 6 (54%) strongly agreed it was easy to understand and comprehensive.

**Table 3:** Extent to which the youth’s toolkit was easy to understand (n=11; comprehensiveness)^a^.

Toolkit components	Strongly disagree, n (%)	Disagree, n (%)	Neutral, n (%)	Agree, n (%)	Strongly agree, n (%)
How and when to ask for accommodations	0 (0)	0 (0)	0 (0)	6 (54)	5 (46)
PowToon video and knowing your rights	0 (0)	0 (0)	2 (18)	5 (45)	4 (36)
Words of advice	0 (0)	0 (0)	2 (18)	4 (36)	5 (54)
Articulate storyline	0 (0)	0 (0)	1 (9)	3 (27)	7 (63)
Simulation scenario 1	0 (0)	0 (0)	0 (0)	4 (36)	6 (54)
Simulation scenario 2	0 (0)	0 (0)	0 (0)	5 (45)	6 (54)
Appendices and resources	0 (0)	0 (0)	3 (27)	2 (18)	6 (54)

^a^Note that numbers are rounded as per journal style.

Table 4 shows the extent to which participants are likely to change their behavior as a result of reviewing the toolkit, where 6 (54%) somewhat agreed, 3 (27%) moderately agreed, and 2 (18%) reported it would change their behavior a lot. Table 5 highlighted the perceived change in behavior after reviewing the toolkit, where 4 (36%) youths plan to disclose their disability to their employer and 2 (18%) might consider it, while 3 (18%) plan to ask their employer for workplace accommodations and 2 (18%) might consider this. Table 6 shows the 2-tailed *t* test results of the pre-post differences in Arc’s self-determination subscales (autonomy, self-realization, and psychological empowerment) and the total scores, all of which showed no statistically significant difference.

**Table 4:** How likely are you to change your behavior as a result of the toolkit? (n=11)^a^.

Extent of agreement	Values, n (%)
Not at all	0 (0)
Somewhat	6 (54)
Moderately	3 (27)
A lot	2 (18)

^a^Note that numbers are rounded as per journal style.

**Table 5:** Perceived change in behavior after reviewing the youth’s toolkit (n=11)^a^.

Response	Plan to disclose your disability to an employer? n (%)	Plan to ask for workplace accommodations, n (%)
Yes	4 (36)	3 (27)
No	1 (9)	0 (0)
Maybe	2 (18)	2 (18)
Already doing this	4 (36)	6 (54)

^a^Note that numbers are rounded as per journal style.

**Table 6:** Pre-post changes for Arc’s self-determination subscales in the youth’s toolkit (n=11).

Subscale	Prestudy score, mean (SD)	Poststudy score, mean (SD)	*t* test (*df*)	*P* value
Autonomy	21.08 (3.87)	21.0 (4.04)	0.22 (11)	.83
Self-realization	5.11 (1.16)	5.00 (0.70)	0.55 (8)	.59
Psychological empowerment	8.66 (0.86)	8.33 (0.70)	1.52 (8)	.08
Total	32.41 (7.91)	33.16 (5.00)	–0.38 (11)	.70

## Discussion

### Principal Findings

This study addressed an important gap in the literature by exploring the initial pilot evaluation of a web-based disability disclosure toolkit for youth with disabilities. Supporting youth to develop such skills is salient because they are an underrepresented and marginalized group in the labor market. Training on this topic could assist with addressing how youth can decide to disclose their condition and request workplace accommodations, with the overall aim of helping young workers to succeed in advocating for their needs as they search for employment.

Our preliminary findings highlight that codeveloping toolkits with youth could help to enhance disability disclosure discussions with their employers [10]. The codevelopment of the toolkit with youth with disabilities highlighted the importance of including knowledge users so that the content was relatable and relevant. Previous research shows that engaging knowledge users is beneficial for the use and uptake of the knowledge and enhancing the impact of the research findings [53]. Related research shows that having a web-based toolkit can be beneficial for youth with disabilities who often have difficulty accessing in-person employment training [54].

Our results demonstrated that participants had positive feedback about the toolkit and suggested some revisions. Such feedback is consistent with other studies showing challenges in the ease of use during pilot-testing of a health care toolkit for adults with autism [55]. Participants in this study also reported that having youth-friendly graphics and plain language and practical case examples developed by youth with disabilities was also beneficial. Our findings are consistent with feedback on other web-based tools for adults with disabilities [56].

Our pilot findings highlight that our toolkit has the potential to inform knowledge or understanding of disability disclosure within the context of employment. Our results also show preliminary evidence that web-based toolkits for youth with disabilities have the potential to enhance perceived confidence about disclosing their disability.

The qualitative findings from our open-ended questions also highlight the usefulness and perceived impact of the tool. Although these results were encouraging, we did not find that our toolkit had a significant impact on self-determination. This finding could be a result of the small sample size for the pilot study that lacked sufficient power to detect change and also that it could take more time to influence change in self-determination behaviors. Other research shows that mentoring could help to improve self-determination, and therefore, future studies should consider adding such an approach to using the toolkit [57]. Aiming to improve self-determination is important for helping youth to optimize their participation and social inclusion in society, such as through employment [58,59].

### Limitations

Caution should be used in interpreting the findings, given the small sample size and that participants were only from one site. It is important to note that we were limited in recruiting participants for the pilot evaluation during the restrictions of the COVID-19 pandemic. Further studies should examine the effectiveness of our toolkit with a larger sample and how any changes in outcomes are maintained over time, in addition to having a control group. This study had an unintentional overrepresentation of females and also a lack of representation from younger age groups (15-19 years), who could benefit from early intervention of employment support. Thus, future research should also consider the impact of sociodemographic characteristics (eg, gender and race or ethnicity) on the impact of the toolkit. Finally, further studies could consider the impact of reviewing the toolkit in person within a group (eg, supported by a mentor or job coach) versus on their own.

### Conclusions

This pilot study provided preliminary evidence of the perceived impact and usefulness of a disability disclosure toolkit for youth with disabilities within the context of employment. Our findings showed how youth with disabilities were involved in codeveloping a toolkit based on their workplace disability disclosure experiences. The qualitative findings emphasized the usefulness of the toolkit and particularly the relatable content, format and design, and perceived impact of the toolkit. Our toolkit shows preliminary potential as an accessible tool to supplement traditional in-person vocational programming for youth with disabilities. Further studies should explore the longer-term impact of this toolkit on employment outcomes with larger samples.

## Appendix

Multimedia Appendix 1 Screenshots of disability disclosure toolkit components.

Multimedia Appendix 2 Table of themes and subthemes for pilot evaluation of a youth's toolkit.

## Abbreviations

REDCap: Research Electronic Data Capture

## Data Availability

The data sets generated and analyzed during this study are not publicly available due to restrictions by our institutional research ethics board. The data could be available from the corresponding author on reasonable request and approval from our research ethics board.
