# Supplementary figures and images for: A Codeveloped Web-Based Disability Disclosure Toolkit for Youth With Disabilities: Mixed Methods Pilot Evaluation
Source: JMIR Form Res. 2023 Dec 8;7:e48609. doi: 10.2196/48609 (PMC10746977; doi:10.2196/48609)

**Multimedia Appendix 1.** Screenshots of disability disclosure toolkit components.


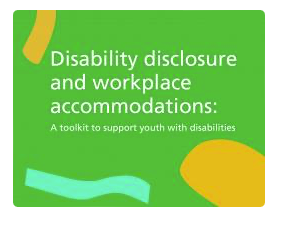


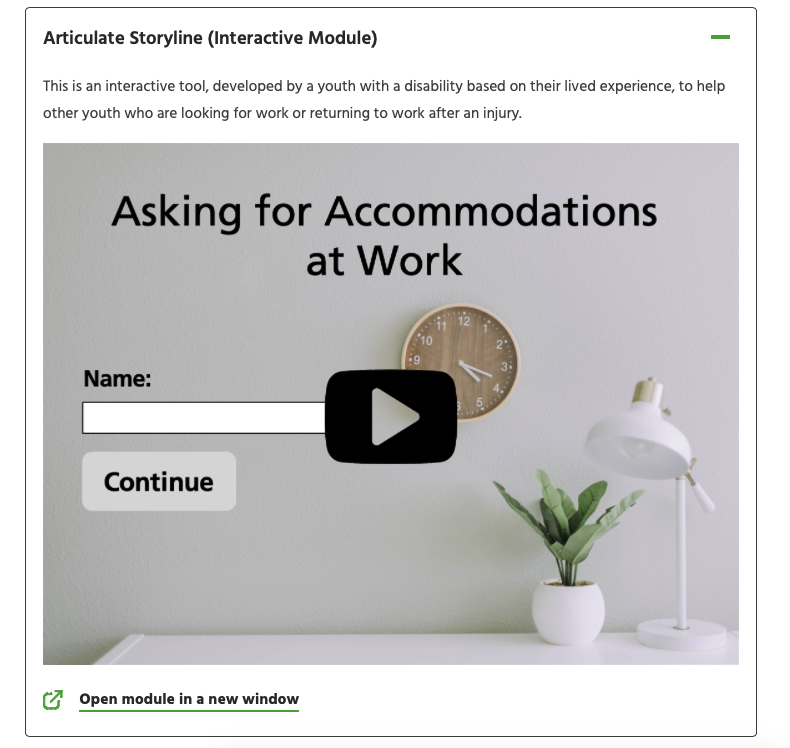


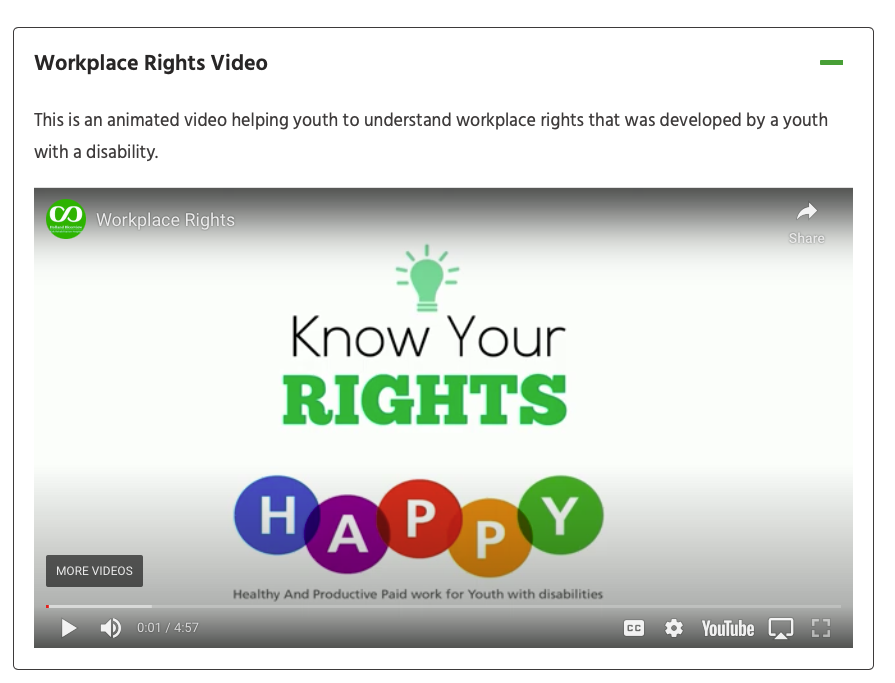

Supplement: Multimedia Appendix 1 [file formative_v7i1e48609_app1.docx]
