# Supplementary material for: A Codeveloped Web-Based Disability Disclosure Toolkit for Youth With Disabilities: Mixed Methods Pilot Evaluation
Source: JMIR Form Res. 2023 Dec 8;7:e48609. doi: 10.2196/48609 (PMC10746977; doi:10.2196/48609)
Supplement: Multimedia Appendix 2 [file formative_v7i1e48609_app2.docx]

**Multimedia Appendix 2.** Table of themes and subthemes for pilot evaluation of a youth’s toolkit.

| **Themes** | **Sub-themes** | **Exemplar quotes** |
| --- | --- | --- |
| Disability disclosure and workplace accommodation experiences |  | -“Don’t make the interview about your disability…it’s almost like a footnote…In many opportunities that puts the employer’s shields down quite a bit too; maybe they find common ground on something like a professional joke or something.” (#1, male with cerebral palsy).  -“With disability disclosure in the past, I haven’t disclosed to my employers and I was just kind of thinking I’ll see how it goes and I didn’t. Usually that wasn’t the best strategy for me. I found that my performance suffered where it was difficult for me because of the cognitive stuff that was going on and at that time it was too late to disclose…I am more comfortable disclosing after I’m hired…so I can see what works best for me with a specific accommodation and say I have a cognitive sensitivity and environmental sensitivity and the employer asks what they can do. I say it’s helpful to have a desk next to a window or to take a 10-minute break every few hours.” (#2, female with acquired brain injury)  -“As somebody with a very visible disability it can be quite awkward not to disclose because, you see it and I see it so it has to be addressed I think. If I don’t address it I feel like I’m kind of avoiding the topic…I tend to ask the employer after the interview if they have any concerns with potentially hiring me so that I can address any doubt they may have in their mind…I don’t want to put them in a position where they’re paying out of pocket or they’re worried that it will be costly for them…most accommodations don’t need that much effort.” (#3, female with cerebral palsy). |
| Usefulness of the toolkit | Relatable content | -“The self-advocacy section was the most useful because this is the most vital skills that someone with a disability can have.” (#5, male with mental health condition)  -“The simulated employer situation where you could choose responses in the conversation was the most useful. I felt like the good responses in that simulation were great templates for me to build conversations off of in the future.” (#9, female with cerebral palsy)  -“The words of advice section that pertains to my visible disability is something I will reference regularly. I will also use the articulate storyline to prepare for conversations with employers. The simulation video makes me more aware of the attitude and mannerisms I am presenting when I interact with others…These sections gave me examples of approaches and language I can use when discussing my disability with employers.” (#7, male with cerebral palsy)  -“I enjoyed the simulations and the disclosure tool” (#14, female with ADHD and learning disability) |
|  | Format and design | -“The color coded tabs and next page buttons made it easier to read a long document and flip back-and-forth content. I like the one page at a time format. It is more like reading a book and does not feel overwhelming with endless scrolling.” (#7, male with cerebral palsy)  -“I liked the videos because I am a visual learner and it is better to see it in action and in a role play” (#6, female with spina bifida)  -“The interactive PDF is helpful because it summarizes all the needed information in one easy-to-access location. The links provide straightforward access to outside resources when more information is wanted…the simulations were fun and interactive.” (#10, male with cerebral palsy)  -“I liked how the topics of each page were bolded to clearly indicate what I was about to read. I like having the tabs on the side too so I could easily switch to a different section for easy access.” (#9, female with juvenile arthritis) |
|  | Suggestions for further development | -“No change, good as is.” (#10, male with cerebral palsy)  -“More of the employer simulated conversations with sample responses would be appreciated.” (#13, female with autism)  -“Shorten it and put more point-form information, especially the important points. Having colors was helpful. Add more examples from other youth sharing their stories.” (#9, female with juvenile arthritis)  -“It would be good to have a words of advice from youths that work for an organization that doesn’t have disability as the primary focus.” (#1, male with cerebral palsy) |
| Perceived impact of the toolkit |  | -“Giving the simulation itself is like a really good boost because, just to see the situation, having worries especially after you’ve gotten the interview and after you’ve gotten the job, you went through all that work then you’re disclosing and asking for accommodations… and you don’t even know how you’re going to say it. So, doing these simulations is enough to have that in the back of your mind, like ok, this is how to do it.” (#2, female with acquired brain injury).  -“It was very informative” (#3, female with cerebral palsy)  -“I will use it as a refresher if I ever need to disclose information.” (#1, male with cerebral palsy).  -“I will keep this toolkit to review before employment” (#8, male with cerebral palsy)  -“I will keep this information in my mind when I am working in my job.” (#6, female with spina bifida)    -“It solidified that employers make unrealistic assumptions about disability in how it impacts our ability to do the job, for some, it can be hard to voice it but it is needed.” (#12, female with cerebral palsy)  -“This information was very helpful! I usually just blurted out my disability when an obstacle comes up because I never knew when to say it. I also learned that my supervisors could have done much more to support me during my time at work.” (#14, female with ADHD and learning disability)  -“I am looking for a paid internship and am having difficulty. This toolkit will help me to develop skills to make it easier.” (#11, female with cerebral palsy) |
